# Trends in Emergency Department Exam Medicare Reimbursements Between 2010 and 2018

**DOI:** 10.7759/cureus.64755

**Published:** 2024-07-17

**Authors:** Summer Ghaith, Zachary Ginsberg, Jordan Pollock, Douglas Rappaport

**Affiliations:** 1 Emergency Medicine, Mayo Clinic, Phoenix, USA; 2 Emergency Medicine, Mayo Clinic Alix School of Medicine, Phoenix, USA

**Keywords:** medical coding, policy, reimbursements, medicare, ed exams

## Abstract

Background: As the Medicare population continues to grow, financial pressure is placed upon hospitals, physicians, and other providers as the payer mix has an increasing proportion of Medicare patients.

Objective: The purpose of this study was to further the understanding of reimbursement trends surrounding the five levels of emergency department (ED) examinations (CPT codes 99281-99285) from 2010 to 2018 and determine how they have changed with respect to each procedure.

Methods: CPT codes were filtered into the 2010 and 2018 Physician/Supplier Procedure Summaries from the Centers for Medicare and Medicaid Services’ website to gather data on emergency physician submissions and Medicare denials and payments.

Results: In 2010, 15,669,196 ED examinations were submitted to Medicare for $7,628,693,382 while in 2018, 16,432,184 ED examinations were submitted for $14,522,456,383. Despite an increase of $397/submission made by emergency physicians, Medicare paid 20.5% of the submitted charges in 2010 for ED examinations and 11.9% in 2018. The denial rate in 2018 was highest for level I ED examinations (11.3%), and the lowest for level V examinations (5.1%). The utilization of level V ED examinations increased 22.3% from 2010 to 2018, while the utilization of the others decreased. Of the five levels of ED examinations, only the level I examination did not exhibit a decrease.

Conclusions: From 2010 to 2018, emergency physicians charged a higher amount for ED examinations, yet Medicare reimbursement accounted for a smaller proportion of these charges, resulting in less payment per submission for the four most common levels. Downward trends in Medicare reimbursement may place financial burdens that could potentially hamper healthcare outcomes.

## Introduction

General trends in reimbursement have shown an overall decrease in reimbursement for healthcare services. This overall decrease in reimbursement has been largely attributed to the Deficit Reduction Omnibus Reconciliation Act of 2005 and the Medicare Access and Children's Health Insurance Program (CHIP) Reauthorization Act of 2015 [1,2]. Currently, United States healthcare billing utilizes Current Procedural Terminology (CPT) codes to categorize procedures and decide reimbursement dollar amounts. Specifically in emergency medicine (EM), studies have shown a decrease in Medicare reimbursement for particular procedures [3,4].

Additionally, recent media has called out EM for "upcoding" procedures, namely, categorizing minor, less serious conditions as serious to upcharge [5]. With these recent controversial allegations coupled with the general decreasing trends in Medicare reimbursement, understanding how reimbursement has changed in the five levels of emergency department (ED) examinations over the years is especially important. As the Medicare population continues to grow, financial pressure is placed upon hospitals, physicians, and other providers as the payer mix has an increasing proportion of Medicare patients. Physicians are under pressure from many different sources, such as patients wanting to maximize their visit length, or hospital administration encouraging shorter visits with patients.

The purpose of this study was to further the understanding of reimbursement trends surrounding the five levels of ED examinations (CPT codes 99281-99285) from 2010 to 2018 and determine how they have changed with respect to each procedure. This study looks at trends in facility price, facility limiting charges, relative value units (RVUs), submitted and denied services, physician-submitted charges, and Medicare-allowed charges.

## Materials and methods

The American College of Emergency Physicians (ACEP)’s top 20 most common ED reimbursement code list was used to analyze ED examination services, of which all five levels of ED examinations (CPT codes 99281-99285) appeared, with levels II-V in the top five. These were then filtered into the 2010 and 2018 Physician/Supplier Procedure Summaries from the Centers for Medicare and Medicaid Services’ website to gather data on emergency physician (provider code 93) submissions and Medicare denials and payments between the years. According to the Department of Health and Human Services (DHHS), approximately 20% of ED visits in 2010 were Medicare patients. In 2018, approximately 24.5% of ED visits were Medicare patients [6].

To review Medicare reimbursement data, we utilized the Physician Fee Schedule Look-Up Tool from the Centers for Medicare and Medicaid Services. We filtered the ED examination levels I-V into the 2010 and 2018 Physician/Supplier Procedure Summaries to gather data on emergency physicians, in particular, by using provider code 93 submissions. Additionally, we abstracted data on Medicare submissions, denials, and payments between the years.

According to the ACEPs, ED examination levels I-V were in the top 20 most common ED reimbursement code list [7]. While there is no national standard for hospital assignment of EM code levels, ACEP provides guidelines for facility billing to categorize ED exams into one of the five levels [8]. The determination of the ED examination level is based solely on "possible interventions" based on the difficulty of procedures [8]. Each level includes the possibility of interventions from the previous level with the addition of other services. Possible interventions for level I examinations include initial assessment, dressing changes, and suture removals [8]. For level II examinations, examples include tests done by ED staff (e.g., urine dip), elastic bandage application, and minor laceration repairs [8]. Level III examination interventions can include receipt of an ambulance patient, nebulizer treatment, and joint aspiration [8]. For level IV examinations, examples of interventions can include the preparation of two diagnostic tests, preparation for X-ray or special imaging, and admission of infusions [8]. Finally, level V examinations can include frequent monitoring of multiple vital signs, administration of blood transfusion, and central line insertions [8].

For each of the five CPT codes (99281, 99282, 99283, 99284, and 99285), the facility price (fee assigned to services provided in facility settings), facility limiting charge (maximum value charged when the provider does not participate in Medicare), RVUs (set with a fee schedule by measuring relative resources, time, and effort used to provide a service), number of services denied, number of submissions, physician submitted charges, and Medicare allowed charges were obtained for each year. The dollar amounts were adjusted for inflation to 2018 USD based on the United States Consumer Price Index (CPI).

To analyze the extracted data, descriptive statistics were used to determine changes, proportions, and total amounts.

## Results

The results are summarized in Table 1. In 2010, 15,669,196 ED examinations were submitted to Medicare for $7,628,693,382. This was equivalent to $487 per submission. In 2018, 16,432,184 ED examinations were submitted for $14,522,456,383. This was equivalent to $884 per submission. Despite the $397 (81.5%) submission increase in charges made by emergency physicians, Medicare decreased their payments of submitted charges from 20.5% of the total submitted charges in 2010 to 11.9% in 2018.

**Table 1:** Comparison of pricing, services, and submissions for levels I-V exams between 2010 (adjusting for inflation) and 2018 RVU: relative value unit

	Facility price	Facility limiting charge	RVU	Number of services denied	Proportion of services denied	Number of submissions	Proportion of total submissions	Total physician-submitted charges	Total Medicare allowed charges	Proportion of total charges allowed	Physician charge per submission	Medicare allowed charges per submission
Level I (99281)												
2010	$23.66	$25.84	0.59	4,776.6	10.5%	45,520.6	0.29%					
2018	$21.60	$23.60	0.6	4,069	11.3%	36,150	0.22%					
Δ Level I	-$2.06	-$2.24	+0.01	-707.6	+0.8%	-9,370.6	-0.07%					
Proportional Δ Level 1	-8.7%	-8.7%	+1.7%	-14.8%	+7.6%	-20.6%	-24.14%					
Level II (99282)												
2010	$46.47	$50.77	1.16	23,816	7.1%	333,653	2.13%					
2018	$42.12	$46.02	1.17	18,210	8.0%	226,633	1.38%					
Δ Level II	-$4.35	-$4.75	+0.01	-5,606	+0.9%	-107,019	-0.75%					
Proportional Δ Level II	-9.36%	-9.36%	+0.86%	-23.5%	+12.7%	-32.1%	-35.21%					
Level III (99283)												
2010	$70.53	$77.06	1.74	154,785	5.8%	2,683,828	17.13%					
2018	$63.00	$68.83	1.75	136,337	7.0%	1,935,659	11.78%					
Δ Level III	-$7.53	-$8.23	+0.01	-18,448	+1.2%	-748,169	-5.35%					
Proportional Δ Level III	-10.68%	-10.68%	+0.57%	-11.9%	+20.7%	-28.9%	-31.23%					
Level IV (99284)												
2010	$133.61	$145.96	3.29	216,689.6	4.9%	4,428,896.6	28.26%					
2018	$119.52	$130.57	3.32	246,783	5.8%	4,235,248	25.77%					
Δ Level IV	-$14.09	-$15.39	+0.03	+30,093.4	+0.9%	-193,648.6	-2.49%					
Proportional Δ Level IV	-10.55%	-10.54%	+0.91%	+13.9%	+18.4%	-4.4%	-8.81%					
Level V (99285)												
2010	$196.67	$214.85	4.78	355,692.7	4.3%	8,177,298.7	52.19%					
2018	$176.04	$192.32	4.89	507,784	5.1%	9,998,494	60.85%					
Δ Level V	-$20.63	-$22.53	+0.11	+152,091.3	+0.8%	+1,821,195.3	+8.66%					
Proportional Δ Level V	-10.49%	-11.71%	+2.3%	+42.8%	+18.6%	+22.3%	+16.59%					
2010 Overall	$94.19 Average	$102.90 Average	2.31 Average	751,318.7	4.79%	15,669,196.9		$7,628,693,382	$1,562,204,525	20.5%	$487	$99.7
2018 Overall	$84.46 Average	$92.27 Average	2.35 Average	913,183	5.56%	16,432,184		$14,522,456,383	$1,728,697,163	11.9%	$884	$105.2
Proportional Δ Overall	-10.3% Average	-10.3% Average	+1.73% Average	+21.5%	+16.08%	+4.87%		+90.37%	+10.66%	-41.95%	+81.5%	+5.5%

In 2010, the denial rate was highest at 10.5% for level I ED examinations, followed by 7.1% for level II ED examinations, 5.8% for level III, 4.9% for level IV, and lastly, it was lowest for level V examinations at 4.3%. The denial rate in 2018 was highest at 11.3% for level I ED examinations, followed by 8.0% for level II examinations, 7.0% for level III examinations, 5.8% for level IV examinations, and the lowest at 5.1% for level V examinations. The utilization of level V ED examinations increased by 22.3% from 2010 to 2018, while the utilization of the other four ED examinations has decreased. Level I examinations decreased by 20.6%, level II by 32.1%, level III by 28.9%, and level IV by 4.4%.

Level V examinations remained the most common ED examination from 2010 to 2018, with 9,998,494 submissions in 2018, and its Medicare payment/submission decreased by about $1 per submission. Level I examinations remained the least common ED examination from 2010 to 2018, with 36,150 submissions in 2018. Of the five levels of ED examinations, only the least common level I examination did not exhibit a decrease in Medicare payment in that time span. Given this decrease, the total payment for all ED examinations was cut by $17,542,385 from 2010 to 2018.

## Discussion

This study found that the number of physician submissions increased between 2010 and 2018, while the proportion of services denied also increased in this time period. Furthermore, the proportion of total charges allowed between 2010 and 2018 also decreased by approximately 42%. Notably, while the utilization of examination levels I-IV decreased, exam level V increased by approximately 17%.

While reimbursement trends are decreasing across specialties [9-11], EM decreases are relevant in particular due to EM physicians being tasked with seeing all patients who present to the ED with an emergent condition to abide by the Emergency Medical Treatment and Active Labor Act (EMTALA) [12]. Therefore, EM physicians cannot choose to only see patients with private insurance, rather than Medicare. Between 2009 and 2018, the proportion of ED visits with patients having private insurance decreased, while those with Medicare increased [13]. This can account for the decrease in income of many physicians after accounting for inflation [14].

Additionally, this study found that the utilization of level V examinations increased, while the rest of the examination levels decreased. Level V examinations have the highest price and RVU as compared to the lower levels, which happened to correlate with the increase in utilization of these examinations. While the controversy over upcoding continues, research suggests that some of the excesses in billing may stem from upcoding [15]. Pressure to upcode may come from hospital leadership as well as insurance payment incentives [15]. This phenomenon may not be a contributor to the changes in CPT coding in this study period but should be considered in light of the pressure placed on EM physicians to bill procedures while Medicare reimbursements continue to decrease.

To our knowledge, this is the first study to specifically look at reimbursement trends specifically within the five levels of ED examinations. We utilized a large public database that grants access to a large number of data. This study has limitations that should be considered. First, this study utilizes only public information for Medicare reimbursement data, which may not be representative of trends in private insurance. However, these trends tend to influence the market as a whole, so it is still practical to analyze these trends in overall reimbursement rates. Second, publicly available Medicare reimbursement data is aggregate data that offers summary statistics on reimbursements rather than all data points available. Therefore, performing statistical analysis of aggregate data is difficult.

## Conclusions

This study provides an analysis of trends in Medicare reimbursement for the five levels of ED examinations. From 2010 to 2018, emergency physicians charged a substantially higher amount for ED examinations, yet Medicare reimbursement accounted for a smaller proportion of these charges, which resulted in less payment per submission for the four most common levels. ED examinations constitute a major service performed by emergency physicians. Therefore, downward trends in Medicare reimbursement may place a financial burden that could potentially hamper healthcare outcomes. Further work must be done to understand the reasoning behind the changes in physician utilization of the various examination levels.

## References

[1] Escarce JJ (1993). Medicare patients’ use of overpriced procedures before and after the Omnibus Budget Reconciliation Act of 1987. Am J Public Health.

[2] Standaert CJ, Smedberg PC (2015). Sustainable growth rate reform is here: should we be happy?. PM R.

[3] Ginsberg Z, Pollock JR, Rappaport DE (2021). Decrease in Medicare reimbursement for single-laceration repairs in the emergency department. Acad Emerg Med.

[4] Pollock JR, Bollig TR, Haglin JM, Sandefur BJ, Rappaport DE, Lindor RA (2020). Medicare reimbursement to physicians decreased for common emergency medicine services from 2000 to 2020. Ann Emerg Med.

[5] Kenen J (2022). Shedding light on upcoding in the ER. Association of Health Care Journalists. July.

[6] (2022). Office of the Assistant Secretary for planning and evaluation, U.S. Department of Health & Human Services. Trends in the utilization of emergency department services, 2009-2018. https://aspe.hhs.gov/pdf-report/utilization-emergency-department-services..

[7] (2022). Topreimbursement codes. American College of Emergency Physicians. https://www.acep.org/administration/reimbursement/top-20-ed-reimbursement-codes/..

[8] (2022). American College of Emergency Physicians; ED facility level coding guidelines; ACEP facility guidelines. https://www.acep.org/administration/reimbursement/ed-facility-level-coding-guidelines/.

[9] Haglin JM, Richter KR, Patel NP (2019). Trends in Medicare reimbursement for neurosurgical procedures: 2000 to 2018. J Neurosurg.

[10] Dominguez JL, Ederaine SA, Haglin JM, Aragon Sierra AM, Barrs DM, Lott DG (2021). Medicare reimbursement trends for facility performed Otolaryngology procedures: 2000-2019. Laryngoscope.

[11] Moore ML, Pollock JR, Haglin JM (2021). A comprehensive analysis of Medicare reimbursement to physicians for common arthroscopic procedures: adjusted reimbursement has fallen nearly 30% from 2000 to 2019. Arthroscopy.

[12] Lindor RA, Ghaith S (2022). Laws of Medicine: Core Legal Aspects for the Healthcare Professional. S.l.: SPRINGER.

[13] (2022). Trends in the utilization of emergency department services, 2009-2018. https://aspe.hhs.gov/sites/default/files/migrated_legacy_files/199046/ED-report-to-Congress.pdf.

[14] Hariri S, Bozic KJ, Lavernia C, Prestipino A, Rubash HE (2007). Medicare physician reimbursement: past, present, and future. J Bone Joint Surg Am.

[15] Geruso M, Layton T (2020). Upcoding: evidence from Medicare on squishy risk adjustment. J Polit Econ.

